# gP for What is Common between Developing Intelligence and Personality: Response to the Commentators

**DOI:** 10.3390/jintelligence6040054

**Published:** 2018-12-06

**Authors:** Andreas Demetriou, George Spanoudis, Hudson Golino

**Affiliations:** 1 Department of Psychology, University of Nicosia, 1700 Nicosia, Cyprus; 2 Department of Psychology, University of Cyprus, 1678 Nicosia, Cyprus; spanoud@ucy.ac.cy; 3 Department of Psychology, University of Virginia, Charlottesville, VA 22903, USA; hfg9s@virginia.edu

**Keywords:** intelligence, personality, development

## 1. Introduction

Three are the main postulates of our article under discussion: First, both human intelligence and personality are hierarchically organized, with a general factor at the apex of each hierarchy, i.e., g for intelligence and GFP for personality. Second, these two general factors interact at various levels, with a common core coordinating this interaction: this is a super-g, i.e., gP (P for the whole Person), connecting g and GFP. Third, everything changes in development; thus, gP changes too. Three studies are presented mapping these changes. Individuals from 7 to 20 years of age were examined, across studies, by a wide range of cognitive and personality measures. To capture these relations, confirmatory factor analysis and structural equation modeling were used, but also other powerful methods currently available. The reader may consult our article for more information about each postulate. This rejoinder focuses on points raised by two commentators, which are crucial for the further development of research and theory in a field that is as old as the inquiry about human nature but rather new as a field of systematic empirical research.

## 2. A “Context of Discovery” 

We concur with Ackerman [1] that the work in our article is a “context of discovery” rather than a “context of justification”. That is, the constructs and ideas presented in the paper form an empirically based frame for hypotheses and predictions about mind–personality relations in development rather than a theory about them. The findings provide a relatively sound starting point for the design and direction of future research that would check them, refine them, or refute them, if necessary. Ackerman notes that the diversity of samples, constructs assessed, and measures weakens the article, because the possible lack of communality of measures undermines generalizations across the studies. We accept the point: These studies were conducted as part of a major ongoing project focusing on the organization and development of the mind. Measures of personality were often collected to examine how personality processes relate to intellectual development. This paper capitalizes on most of these studies. 

Therefore, a next wave of research may start from here that would ad hoc measure the constructs of interest and predictively model their relations. Future research would have to devise measures of cognitive and personality processes at equivalent levels of precision and construct validity to minimize the concerns of the commentators, which we do share. The main difference between cognitive and personality measures lies in what they stand for: cognitive measures stand for measures of actual performance as specified by the researcher; personality measures stand for self-characterizations given by the research participant. Each set of measures may be internally highly reliable, as observed here; however, they may still reflect different levels of precision and objectivity in the measurement of the constructs they represent. This is especially true if children and adolescents are involved, because one’s own performance is differentially transparent to self-observation and evaluation during growth. 

## 3. “Smite, but Hear Me” 

Bäckström’s [2] objections are epistemologically interesting, and we are grateful that he offers us the opportunity to clarify several important points. First, it seems to us that Bäckström approaches the research presented in our article from the point of view of his own research that is critical of the hierarchical model of personality and the GPF as such (see references in Bäckström’s commentary). He himself conducted research trying to show that hierarchical organization of personality and the GFP may be ruled out as artefacts of the personality inventories used and the modeling methods employed. The stance against hierarchical organization and general factors is as old as empirical psychology. Noticeably, it started in the psychology of intelligence itself. Prominent examples are classical theories, such as Thurstone’s [3] theory of primary abilities, or more current theories, such as Gardner’s [4] theory of multiple intelligences. Cognitive-differential psychology is already beyond this dispute. Hierarchical models of the mind involving specific and general abilities dominated and are here to stay. Notably, modern brain [5] and genetic research [6] came in strong support of the hierarchical model of the mind. Perhaps, a brain g and a genetic g underlie the psychometric g [7]. It may be time for brain and genetic research to search for gP as well. Admittedly, however, this study is only at its beginning; genes related to cognition or personality account for a very small part of the variance, and findings are often inconsistent [8]. 

Before this target article was written, we were neither in favor nor against the GFP. The findings came in support of it. In fact, motivated by the reviewing process, we systematically tested models to disconfirm both the GFP and the gP. This has not been possible: Both constructs persisted to emerge across studies; these models are described in the paper. In fact, these findings align with several other papers published in this Special Issue, which showed that cognition–personality relations emerge at different levels of the organization of each realm and engage several components in each [9,10,11]. Having said this, we would not object that the tools used may have contributed to finding these constructs. However, it is a matter of a priori specifically designed research to further examine these constructs. We believe that a part of Bäckström’s difficulty in accepting these constructs comes from his non-developmental approach to psychological research (see references in Bäckström’s commentary). This is clear in his taking differences in developmental trends of personality processes across studies as a sign of inconsistency rather than as what they are: developmental differences reflecting age differences between studies. We showed in several other studies that the relations between important constructs of intelligence, such as speed, attention control, working memory, reasoning, and awareness, change systematically with the developmental cycle, reflecting the developmental reconstruction of representational and processing possibilities in each cycle [12,13]. The present studies suggest that it is worth examining if cognition–personality personality relations may also change with development rather than dismissing them as artefacts of the testing or modeling methods. 

Physics progresses by first assuming and then searching for and experimentally demonstrating “entities of nature” to account for phenomena unaccounted for by the current models. The notorious Higgs particle is an example of a big recent discovery in the search of general forces of nature. We suggest that g, GFP, and gP may be such “entities of nature”. They seem needed to account for patterns in our experimental data but also for consistent patterns of behavior of real people, be they infants, children, or adults, especially as they change over age. For instance, gP may explain the distinct profile of individuals reflected in general characterizations addressed to them, such as strong, determined, persistent, flexible, resilient, etc. Obviously, both cognitive and personality properties converge and merge into these characterizations. We suggested that cognizance is a core process in gP, the junction bridging g with GFP. This process changes drastically in development, causing changes in g–GFP relations. The three studies presented in the article provide a glimpse of these relations. For instance, they show that likeability, a phenomenon Bäckström invokes to back his argument, is actually a derivative of changes in this construct. Notably, evidence suggests that in one-factor models of personality an *Evaluation* factor dominates [14]. This is a desirability dimension defined by personal qualities ranging from socially desirable to non-desirable. It reflects the ability to evaluate one’s own value with respect to the group. This is also the first factor emerging in cognitions of young children. This aligns well with our findings showing this dimension to be strongly developmental. Thus, these studies invite for a systematic examination of the interaction between this self-evaluation dimension and the accuracy in self-representation and self-presentation.

Bäckström raised several points about some technical issues in our modeling approach. First, he notes that Exploratory Graph Analysis (EGA) is not confirmatory. Precisely: EGA was used to map the terrain of a not-so-well-known territory and capture relationships between a theory-based structure and the empirical structure that is estimated from the data. Then, the exploratory model was verified via confirmatory and structural equation modeling. In this sense, the use of EGA in the paper adds value to the main goal of the paper, since it enables the comparison of a theory-based model with a data-driven model. 

Second, Bäckström takes some pains to show that the direct Gf–GFP relation in the third study is negative (−0.16), while the indirect relation estimated from the Gf–cognizance (0.22) and the cognizance–GFP relation (0.58) is positive and lower (0.13). This is not a problem. On the one hand, some personality dimensions, such as neuroticism and conscientiousness, are negatively related to g (see also [15]). Thus, when these dimensions dominate in GFP, the g–GFP relation would come out negative. On the other hand, cognizance, the mediator, is positively related to each of these two dimensions because it is highly self-representational, like personality, and reflective of Gf variations. Therefore, using these two dimensions to derive the Gf–GFP relation misses the negative relations mentioned above. This is the case in Study 3. Additionally, the presence of academic measures in that specific model changes the balance of the cognitive–personality relations, as they themselves are largely cognitive measures. Therefore, local calculations as those reported in the commentary miss the interactive part of multivariate models.

Finally, Bäckström is concerned that very high relations between cognitive and personality dimensions, such as those reported across studies, may reflect model mis-specification. Model mis-specification is always a risk in structural equation modeling, especially in research lying in the “context of discovery”, where theory for generating specification predictions is not fully precise. However, our models were technically acceptable, as indicated by the technical information provided by the model outputs (see Supplementary Tables). The values obtained reflect the fact that we used very refined developmental measures of cognitive processes, obtaining the variation that is necessary to map their relations with personality measures. Finally, it is stressed that we residualize predictor factors in the various models (see models shown in the Supplementary). This ensures that collinearities are controlled. To our knowledge, few programs, such as EQS, can do this with precision. Thus, if a precise residualization of factors was not done by Bäckström, his models are not comparable to our models. 

In short, technical and other problems may always linger in this kind of modeling blurring boundaries between actual psychological constructs and technical constructs or distorting their relative importance [16,17]. However, the signs emerging from our research remind us of Themistocles call, about 2500 years ago, to the opponent of his strategy: “Smite, but hear me”! The opponent listened, and they won. In the present context, the research presented in our article suggests that it is in the interest of science to intensify exploration of these constructs rather than dismiss them as artefacts. With caution suggested by our thoughtful commentators, exploring these constructs will enhance our understanding of cognition–personality relations in development.
